# The Nose–Lung Axis in Chronic Obstructive Pulmonary Disease: An Emerging Interface for Clinical Endotyping, Biomarker Discovery, and Integrated Management

**DOI:** 10.3390/diagnostics16071035

**Published:** 2026-03-30

**Authors:** Pasquale Ambrosino, Giuseppina Marcuccio, Claudio Candia, Fabio Manzo, Christian Basile, Guido Grassi, Mauro Maniscalco

**Affiliations:** 1 Istituti Clinici Scientifici Maugeri IRCCS, Scientific Directorate of Telese Terme Institute, 82037 Telese Terme, Italy; 2 Istituti Clinici Scientifici Maugeri IRCCS, Pulmonary Rehabilitation Unit of Telese Terme Institute, 82037 Telese Terme, Italy; giuseppina.marcuccio@icsmaugeri.it (G.M.); claudio.candia@icsmaugeri.it (C.C.); 3 Fleming Clinical Laboratory, 81020 Casapulla, Italy; fabioce26@gmail.com; 4 Department of Clinical Science and Education, Karolinska Institutet, 17177 Stockholm, Sweden; christian.basile@ki.se; 5 ANMCO Research Center, Heart Care Foundation, 50121 Florence, Italy; 6 Department of Medicine and Surgery, Milano-Bicocca University, 20126 Milan, Italy; guido.grassi@unimib.it; 7 Department of Advanced Biomedical Sciences, University of Naples “Federico II”, 80131 Naples, Italy

Chronic obstructive pulmonary disease (COPD) continues to be framed predominantly as a disorder of the lower airways, defined operationally by airflow limitation, and assessed through a combination of spirometry, symptom burden, and exacerbation history. This framework remains clinically useful, but it does not fully account for the substantial heterogeneity observed in symptom burden, exacerbation risk, inflammatory signatures, and response to inhaled corticosteroids (ICS) [1]. The dissociation between lung function and patient-reported outcomes, together with the inconsistent predictive performance of blood eosinophils, shows how current diagnostic tools capture only part of the underlying biology of this disease [2,3]. To advance COPD toward genuine precision medicine, its diagnostic structure should expand from a single anatomical focus to an integrated airway perspective. This shift invites reconsideration of the respiratory tract as a continuous biological surface rather than a series of isolated compartments.

The anatomical continuity of the respiratory tract from the nose to the alveoli has been recognized since antiquity. As early as the second century AD, Claudius Galenus described the nose as a respiratory organ and acknowledged the paranasal sinuses as part of the airway system [4]. Despite this longstanding recognition, the upper airway remains largely excluded from structured diagnostic assessment in COPD.

The upper airway represents the first epithelial interface exposed to inhaled particles, pathogens, and pollutants [5]. In COPD, however, it is rarely incorporated into structured diagnostic assessment [6], and this omission has conceptual and methodological implications. The nasal and sinonasal mucosa constitute an immunologically active surface where inflammatory polarization, oxidative stress, and microbial perturbations can be repeatedly sampled with minimal invasiveness [7]. Few compartments in respiratory medicine combine accessibility, repeatability, and biological richness to the same extent, and, from a diagnostic standpoint, such a combination is difficult to ignore.

The upper airway also represents the primary site of entry for respiratory pathogens implicated in COPD exacerbations. Experimental rhinovirus infection studies have shown higher viral loads and enhanced airway inflammatory responses in COPD patients compared with controls, with evidence of cross-compartment inflammatory coherence between nasal and lower airway compartments [5,8]. Moreover, cigarette smoke and environmental pollutants induce oxidative stress in the nasal epithelium through molecular pathways that overlap with those operating in the bronchial mucosa [9]. Components such as acrolein have been shown to promote pro-inflammatory responses in primary nasal epithelial cells [10], reinforcing the concept that the sinonasal mucosa actively contributes to smoke-related injury rather than serving as a passive conduit.

In this scenario, it is worth noting that upper airway symptoms are highly prevalent in COPD, reported in up to 88% of patients with moderate-to-severe disease, although they remain insufficiently addressed in routine clinical practice [11]. In keeping with this, population-level data from the Swedish SCAPIS cohort, which included more than 30,000 individuals, demonstrated associations between chronic rhinosinusitis (CRS), chronic airflow limitation, and lower respiratory symptoms in both ever smokers and never smokers [12]. Chronic nasal symptoms, whether defined as chronic rhinitis or CRS, have also been shown to independently impair quality of life and to be associated with greater dyspnea burden and higher COPD symptom scores [13,14]. A retrospective cohort study of over 2000 COPD patients further demonstrated that comorbid chronic rhinitis was significantly associated with increased risk of 30-day hospital readmission, suggesting that upper airway inflammation represents a clinically relevant component rather than an incidental finding [15]. Importantly, CRS itself has been independently associated with significant activity, work, and social limitations at the population level, with an estimated 11.5 million aggregate workdays lost annually in the United States alone [16], and these functional consequences may compound the exercise intolerance, physical disability, and rehabilitation needs that already characterize COPD as a systemic disease [17,18,19,20]. Despite this growing evidence, CRS remains substantially underdiagnosed in COPD. In a clinical study of 222 patients with COPD, up to 82% of CRS cases had not been recognized prior to systematic otorhinolaryngological evaluation, although affected individuals exhibited significantly worse health-related quality of life [6].

However, prevalence alone does not justify systematic integration of the upper airways into diagnostic pathways of COPD. The central issue is not whether rhinitis coexists with COPD, but whether the sinonasal compartment provides biologically meaningful information for patient stratification. The “united airways” model may offer a coherent mechanistic framework to address this question [21]. In CRS with nasal polyps (CRSwNP), concordant inflammatory signatures in nasal polyp tissue and bronchial mucosa support immune alignment across airway compartments [22]. COPD has traditionally been viewed as predominantly neutrophilic and relatively steroid resistant, but it is now clear that a substantial proportion of patients exhibit a type 2 inflammatory signature and show greater responsiveness to ICS [23,24]. Lung tissue analyses have further refined this paradigm, as gene-set enrichment analysis of bronchial brushings from patients with mild-to-moderate COPD has demonstrated significant activation of interleukin (IL)-4 and IL-13 signaling pathways, but not IL-5-related pathways, in association with tissue eosinophil presence [25]. Moreover, higher expression of IL-13-responsive genes, including POSTN and CCL26, has been associated with greater bronchial eosinophil density, reinforcing the concept that IL-13-driven pathways contribute directly to local eosinophil recruitment within the bronchial mucosa [25]. This apparent dissociation at the transcriptomic level does not exclude a broader contribution of IL-5 to eosinophilic airway inflammation, as IL-5 is known to promote eosinophil maturation, activation, and survival through mechanisms that may not be fully captured by tissue-level gene-set enrichment analysis [26]. These same cytokine axes are well-established drivers of type 2 inflammation in CRSwNP, reinforcing the biological plausibility that inflammatory polarization may extend across the upper and lower airways rather than remaining compartmentalized [27].

If inflammatory polarization extends along the respiratory tract rather than remaining confined to the bronchial tree, then the sinonasal compartment cannot be considered biologically irrelevant. In that context, structured sinonasal phenotyping in COPD patients with elevated eosinophil counts becomes a logical diagnostic step rather than a theoretical extension. Nasal cytology provides a reproducible method for cellular characterization, distinguishing eosinophilic, neutrophilic, mast cell predominant, and mixed inflammatory patterns [28]. In upper airway disease, endotype-driven approaches have already shown that such stratification can inform therapeutic decision-making [29]. Within COPD, standardized nasal cytological assessment could serve as a low-burden adjunct to systemic biomarkers, identifying inflammatory polarization aligned with or divergent from peripheral blood eosinophilia. Of course, this approach requires prospective validation, but it remains biologically plausible and relies on diagnostic techniques already established in sinonasal disease.

Biochemical mediators may further support cross-compartment interrogation. Sputum IL-8 correlates with inflammatory burden and disease characteristics in COPD [30], and comparable chemokine networks can be detected in nasal secretions in inflammatory airway disorders [31,32]. Oxidative stress signatures follow a similar pattern. Leukotriene B4 and 8-isoprostane increase in exhaled breath condensate during COPD exacerbations [33], and analogous eicosanoid profiling has also been documented in nasal lavage fluid in CRS [34]. These observations do not imply equivalence between nasal and bronchial sampling, but they indicate that other accessible airway compartments may host quantifiable mediator networks intersecting with pathways implicated in COPD pathobiology [35].

If mediator networks relevant to COPD can be detected across airway compartments, the central issue shifts to clinical relevance. The nasal compartment offers an accessible and repeatable site for biological sampling. Its role, however, should not rest on mirroring bronchial pathology but on whether it meaningfully refines patient stratification within existing clinical frameworks. Any proposal for integration should therefore align with the treatable traits paradigm, for which emerging data provide a preliminary rationale [36].

COPD patients with concomitant upper airway disease have demonstrated greater eosinophilic features and increased lower airway symptom burden [37]. In parallel, peripheral blood eosinophils have been progressively incorporated into therapeutic decision-making, and meta-analytic data support exacerbation reduction with ICS in patients with elevated eosinophil counts [23]. Broader analyses position eosinophilia as a candidate treatable trait while underscoring the need for more refined implementation strategies [24]. Within this context, structured sinonasal phenotyping may complement systemic biomarkers, particularly in individuals with intermediate or fluctuating eosinophil levels, provided that prospective studies demonstrate additive predictive value. Moving from biological plausibility to clinical applicability, however, requires a clearly defined methodological pathway.

From a practical standpoint, validated patient-reported instruments such as the 22-item Sino-Nasal Outcome Test (SNOT-22) [38] can be readily integrated into routine COPD assessment to screen for sinonasal comorbidity, while nasal cytology, a low-cost office-based technique already standardized in rhinology, could complement blood eosinophil counts by providing direct information on local inflammatory polarization. Whether identification and treatment of upper airway disease translate into improved COPD outcomes remains to be established, but the diagnostic tools required for this assessment are already available in clinical practice. Obviously, any extension beyond cytology should remain hypothesis-driven rather than exploratory. Targeted interrogation of the sinonasal compartment should focus on pathways already implicated in bronchial disease, for instance, type 2 inflammatory signaling, and employ selected measures, such as cytokine profiling of nasal secretions or functional readouts like nasal nitric oxide, only when aligned with a predefined biological question. Prospective cohort studies should then determine whether such sinonasal variables show independent and clinically meaningful associations with exacerbations, treatment response, or longitudinal lung function decline, as integration into practice is justified only if empirical added value is demonstrated. Specifically, prospective cohort studies incorporating systematic sinonasal phenotyping alongside established COPD biomarkers are needed to determine whether nasal cytological patterns or mediator profiles add independent predictive value for exacerbation risk or treatment response. In parallel, interventional trials evaluating whether targeted management of concurrent upper airway disease reduces exacerbation frequency or improves health status in COPD would provide the clinical evidence required to justify integration into routine practice.

In conclusion, COPD originates with inhaled exposure and epithelial contact, with the first immunological interface being nasal. If precision medicine in COPD is to rely on measurable, reproducible, and biologically coherent signals, the upper airway should not remain diagnostically peripheral. Its role will ultimately be defined by evidence showing that structured evaluation of the sinonasal compartment enhances stratification or therapeutic targeting beyond current standards.
